# Emotion Recognition and Perspective Taking: A Comparison between Typical and Incarcerated Male Adolescents

**DOI:** 10.1371/journal.pone.0170646

**Published:** 2017-01-25

**Authors:** Larisa Morosan, Deborah Badoud, Alexandra Zaharia, Tobias Brosch, Stephan Eliez, Anthony Bateman, Patrick Heller, Martin Debbané

**Affiliations:** 1 Developmental Clinical Psychology Unit, Faculty of Psychology and Educational Sciences, University of Geneva, Geneva, Switzerland; 2 Developmental Imaging and Psychopathology Lab, Office Médico-Pédagogique, Department of Psychiatry, University of Geneva, Geneva, Switzerland; 3 Swiss Center for Affective Sciences, University of Geneva, Geneva, Switzerland; 4 Consumer Decision and Sustainable Behavior Lab, Faculty of Psychology and Educational Sciences, University of Geneva, Geneva, Switzerland; 5 Research Department of Clinical, Educational and Health Psychology, University College London, London, United Kingdom; 6 Anna Freud Centre, London, United Kingdom; 7 Department of Forensic Medicine and Psychiatry, Department of Communitarian, Emergency and First Aid Medicine and Psychiatry, Department of Mental Health and Psychiatry, University Hospital of Geneva, Geneva, Switzerland; Institut d'Investigacio Biomedica de Bellvitge, SPAIN

## Abstract

**Background:**

Previous research suggests that antisocial individuals present impairment in social cognitive processing, more specifically in emotion recognition (ER) and perspective taking (PT). The first aim of the present study was to investigate the recognition of a wide range of emotional expressions and visual PT capacities in a group of incarcerated male adolescents in comparison to a matched group of community adolescents. Secondly, we sought to explore the relationship between these two mechanisms in relation to psychopathic traits.

**Methods:**

Forty-five male adolescents (22 incarcerated adolescents (*M*age = 16.52, *SD* = 0.96) and 23 community adolescents (*M*age = 16.43, *SD* = 1.41)) participated in the study. ER abilities were measured using a dynamic and multimodal task that requires the participants to watch short videos in which trained actors express 14 emotions. PT capacities were examined using a task recognized and proven to be sensitive to adolescent development, where participants had to follow the directions of another person whilst taking into consideration his perspective.

**Results:**

We found a main effect of group on emotion recognition scores. In comparison to the community adolescents, the incarcerated adolescents presented lower recognition of three emotions: interest, anxiety and amusement. Analyses also revealed significant impairments in PT capacities in incarcerated adolescents. In addition, incarcerated adolescents’ PT scores were uniquely correlated to their scores on recognition of interest.

**Conclusions:**

The results corroborate previously reported impairments in ER and PT capacities, in the incarcerated adolescents. The study also indicates an association between impairments in the recognition of interest and impairments in PT.

## Introduction

Antisocial behavior is characterized by the violation of social norms and the rights of others [1]. Individuals characterized as being antisocial constitute a heterogeneous group, and previous research has conceptualized their behavior in different ways, using terms such as *aggression* and *delinquency*. When such behaviors meet diagnostic criteria, a number of psychiatric diagnoses might apply (e.g., conduct disorder, oppositional defiant disorder or antisocial personality disorder). Research into the personality basis of antisocial behavior suggests that psychopathy, defined by lack of affectivity, a deceitful interpersonal style, and impulsive and irresponsible behavior, might foster the development of antisocial behavior, being associated with more severe, aggressive, and stable antisocial behaviors [2,3]. The developmental trajectories of antisocial youths are marked by serious personal, social, and educational challenges, and the damage resulting from their behaviors and the necessary means to regulate them incur notable costs to society [4]. Manifestations of antisocial behavior during adolescence might represent precursors of more serious antisocial problems in adulthood [5]. Thus, investigating the factors implicated in the early manifestations of antisocial behavior could contribute with crucial information toward a better understanding of these behaviors, and might inform early prevention and intervention strategies.

For this purpose, several studies have explored the psychological processes in which impairments might sustain antisocial behaviors. Social impairments represent the most notable core problem in antisocial populations, thus, a large body of research has focused on socio-cognitive processes implicated in antisocial behaviors [6]. Among the socio-cognitive processes associated with antisocial profiles, impairments in the encoding of emotional information and in understanding others’ mental states have consistently been found in antisocial youths and adults [1,7–9]. Emotion recognition (ER) and perspective taking (PT) represent two of the most important processes sustaining social behavior, leading some authors to hypothesize that the impairments in these two processes might sustain the impairments in social cognitive functioning reported in antisocial individuals [10].

To look at this issue more closely, contemporary theories aim to describe the relationship between psychological processes that underlie social cognitive impairments in antisocial populations. One approach postulates that antisocial individuals have impairments in the recognition of specific emotions, namely, a reduced recognition of fear and sadness [11–13]. Blair [14] hypothesized that the natural human capacity to recognize distress cues in others, such as facial expressions of fear and sadness, provides a strong inhibitory effect on any prior violent intention or action aimed to harm them. However, given antisocial individuals’ impairments in the recognition of these two emotions, these individuals would not always have the inhibitory effect provided by this mechanism at their disposal, and would therefore engage in more frequent antisocial actions. This theory has been supported by several studies showing impairments in the recognition of fear and/or sadness in incarcerated adolescents and adults [12,15–18]. Studies conducted in the general population on children, adolescents, and adults with high psychopathic traits have contributed supporting evidence showing impaired recognition of specific emotions [13,19–24].

Beyond these results, other authors have suggested that antisocial populations show impairments in the recognition of a wider range of emotions [7]. Impaired recognition of anger, disgust, happiness, and/or surprise has been observed in incarcerated adolescents and adults [17,18,25–27]. These results were further reported in children with conduct disorder [23] and in children, adolescents, and adults with high psychopathic traits from the general population [20,28]. More recently, Sharp, Vanwoerden, Van Baardewijk, Tackett, and Steege [29] postulated that antisocial individuals might primarily present impairments in the recognition of complex emotional stimuli. They reported that children from the general population scoring high on psychopathic traits had impairments in the recognition of complex emotions–impairments that are more strongly correlated with the callous-unemotional dimension of psychopathy. Additionally, Dolan and Fullam [30] reported impairments in the recognition of complex emotions in non-psychopathic incarcerated adults. Complex emotions could be theorized as social emotions, in that they imply an additional inference on more subtle mental states of others [31,32]. Thus, impairments in the recognition of complex emotions may be somehow tied to impairments in PT, which represents an important factor sustaining the inference of psychological stance in others [33].

The capacity to take into consideration the perspective of others and to distinguish it from one’s own is a crucial mechanism that sustains a successful social adaptation [34]. The basic PT abilities develop during childhood, however more complex dimensions of PT continue to specialize during adolescence and early adulthood [35]. Several studies have reported that adults and adolescents fail to use the perspective of others during social interactions [34,36]. No study, to our knowledge, has yet investigated visual PT in incarcerated populations. However, some research focused on cognitive PT in antisocial populations. In incarcerated adults, some studies reported impairments in inferring cognitive mental states, such as intentions, in others [17,37], but not all studies found this pattern [30,38,39]. Only one study [40] investigated cognitive PT abilities in a sample of children and adolescents with antisocial behavior and autism spectrum disorder, and failed to observe any impairments in relation to antisocial behaviors. It is important to note that previous studies employed assessment measures that were not optimally sensitive to the developmental aspects of PT. These tasks often produce ceiling effects when used in adult and adolescent populations [33]. In this context, the examination of PT skills in incarcerated youths using a paradigm sensitive to the later stages of social-cognitive development appears to be warranted.

On the basis of the studies reviewed above, it appears that methodological limitations in both ER and PT paradigms may currently limit our understanding of the implications of deficits in ER and PT for early antisocial behaviors. In this study, we aimed to overcome some of these limitations.

First, the majority of studies investigating ER used only the six basic emotions, presented almost exclusively as still facial stimuli. Only one study used body postures [41] and a few others have used emotional vocal stimuli [22,42,43]. Because the ER stimuli tap only one perceptual modality, the ecological validity of the ER tasks used to date may limit their generalizability to other antisocial samples and to real-life situations. In order to increase ecological validity when examining ER, we employed the Geneva Emotion Recognition Task (GERT), which presents a wide range of emotions, portrayed by facial, vocal and postural expressions, in dynamic video clips. Extending the range of emotions and using dynamic stimuli from different modalities allows investigation of ER in a way that more closely replicates how it occurs in everyday life [44].

With regard to tasks investigating PT abilities, the studies focusing on the cognitive PT in antisocial populations employed tasks with little sensitivity for the developmental aspects of PT during adolescence. In the present study, we employed a task that is recognized to be sensitive to development of perspective-taking during adolescence, the “Director task” [36]. This task requires from the participants to account for the perspective of another person giving instructions to perform the task properly. The task involves a procedure that distinguishes PT performance from performance dependent upon executive functions.

Finally, no study, to our knowledge, has investigated ER and visual PT simultaneously in antisocial adolescents, meaning that the potential interplay between these social-cognitive functions has not been tested. We were further interested in investigating the relationship between ER and PT, and between these two capacities and the dimensions of psychopathy, in a group of incarcerated adolescents, as well as a group of typically developing adolescents. We focused on a group of incarcerated adolescents as it is believed that delinquency represents a severe form of antisocial behavior, and that adolescence is a critical period for its emergence [3]. Based on the previous studies, our hypotheses were that (1) in comparison to a community group of adolescents, the incarcerated adolescents would have deficits in recognizing a wide range of emotions; (2) the incarcerated adolescents would also show impairments in PT abilities; (3) impairments in ER would be positively correlated with impairments in PT; and (4) the ER and PT impairments would be positively correlated with a self-reported measure of psychopathy.

## Materials and Methods

### Sample

Twenty-two male incarcerated adolescents (IA) (*M*age = 16.52, *SD* = 0.96) from an observation and detention center for youths in Geneva, Switzerland, took part in the study. Twenty-three male community adolescents (CA) with no previous criminal convictions, matched for age to the IA (*M*age = 16.43, *SD* = 1.41), formed the comparison group. The community adolescents were recruited via advertising leaflets and by word of mouth.

The inclusion criteria were male gender, age 13–19 years, and fluency in French. Information about the reason for incarceration was available for only 20 of the IA; for the majority, this was having committed more than one criminal offense, including physical and verbal aggression (55%), substance abuse (45%), theft (30%), robbery (15%), runaways from home (15%), and driving violations (10%). Eighteen adolescents from the IA group were screened for psychiatric problems according to DSM-IV criteria using the Kiddie-SADS Present and Lifetime Version (K-SADS-PL) semi-structured interview [45]. Table 1 shows the diagnostic information for the IA group, as well as the ethnic composition of both groups.

**Table 1:** Demographic and diagnostic information of the sample.

Variable	Community adolescents (% of the group)	Incarcerated adolescents (% of the group)
*Age (above 18 years old)*	*4*.*3%*	*4*.*5%*
*Ethnic origin*		
Swiss	58.3%	36.4%
Mixed	8.3%	18.2%
Southern European	4.2%	36.4%
Eastern European	16.7%	4.5%
South American	-	4.5%
African	4.2%	-
*K-SADS-PL diagnostics*		
Conduct disorder	-	22.2%
Substance abuse	-	22.3%
Conduct disorder and substance abuse	-	16.8%
Substance abuse and other comorbidities (ADHD, MDD, panic disorder)	-	11.2%
No diagnosis	-	22.3%

K-SADS-PL, Kiddie-Sads Present and Lifetime Version; ADHD, attention-deficit/hyperactivity disorder; MDD, major depressive disorder.

Written informed consent was obtained from all the participants and, for participants under 18 years old, also from their parents. The protocol was approved by the Institutional Review Board of the Department of Psychiatry of the University of Geneva Medical School. The adolescents in both groups received monetary compensation for their participation in the study (30 CHF).

### Measures and Instruments

#### Emotion recognition

The GERT [46] was used to evaluate the participants’ ER abilities. The GERT is a computer-based task that presents 82 videos, each lasting between 2 and 4 seconds. The videos feature 10 adult actors (five female and five male, all Caucasian) exhibiting an array of 14 emotions (labeled as fear, anxiety, sadness, despair, surprise, disgust, joy, amusement, interest, irritation, anger, pleasure, pride, relief) using congruent gestural, facial, and vocal (one or two pseudo-linguistic sentences) expressions. Before the task began, the participants were instructed to read the definitions of the labels for all the emotional expressions and were presented with three training examples. A glossary providing the definitions of each emotion was at the participants’ disposal for the duration of the task. Each emotion was presented six times, with the exception of despair, which was presented five times. The videos were randomized across participants. Each video could be viewed only once, and after each video, the names of all the emotions were displayed on the computer screen. The videos were presented on a laptop, using Adobe AIR. The participants were instructed to choose, using the computer’s mouse, the label for the emotion they believed the actor had expressed. The labels were presented until a response was made; only one choice was possible for each video.

For each participant, the accuracy recognition score for each emotion was calculated following Schlegel et al. [47], using the unbiased hit rate, which limits the biases toward certain response categories. The unbiased hit rate for an emotion is calculated as the squared frequency of correct responses for that emotion, divided by the product of the number of presentations of the emotion and the overall frequency of choosing that emotion.

#### Perspective taking

A French-language adaptation [48] of Dumontheil et al.’s [36] computerized paradigm, the Director task, was used to evaluate participants’ visual PT abilities. The task included two conditions, characterized as the Director and No-Director, which included experimental, control, and filler trials. In the Director condition, a director stood behind the shelf and asked the participant to select and move specific objects. In the No-Director condition, the instructions were identical, with the exception that they did not come from the director, but required the participant to follow a rule, ignoring objects placed in grey slots. This condition has been designed to match the cognitive processes engaged in the director condition, such as working memory, rule following or inhibition of the prepotent response. Experimental trials involved, in the Director condition, taking the perspective of the director and, in the No-Director condition, following the rule, while ignoring the distractor (an object that better fit the instructions, but could not be visible for the director or was placed in a grey slot). In the control trials, the disposition of the objects on the shelves was the same as in the experimental trial, except that the distractor was replaced by an irrelevant object (an object that didn’t match the target object). In the filler trials, all the objects could be seen by both the director and the participant. Each condition (Director and No-Director) contained 8 experimental, 8 control and 48 filler trials. The order of trials was counterbalanced between the participants. Standardized instructions were given to the participants. For the Director condition, an example was shown to the participants, emphasizing the differences between their perspective and the director’s perspective, explaining that they should take the perspective of the director when they move the object indicated. In addition, the participant had to show an object that they and the director could see, and an object that only they could see and to perform an example trial. Before the Non-Director condition, another set of instructions was presented to the participants. It was explained that the instructions refer only to the objects in the grey slots. The participants had to show an object in a grey slot and one in a transparent slot, and to perform an example trial. All the participants performed correctly the example trial of each condition before they begun the task. The Director condition was always presented before the No-Director condition.

Stimuli consisted of 16 slot shelves arranged in a 4×4 grid, containing a total of eight different objects. For each shelf-object configuration, the participants had to perform three different trials, two filler and one control or one experimental trial. The response had to be given using a computer mouse in 3.6 seconds. The task would continue even if no response were given. Each condition lasted for approximately 5 minutes. The task was designed using E-Prime, version 2.0 (Psychology Software Tools, Inc.) and it was presented on a laptop. For a complete description of the task, see Dumontheil et al. [33]

In the present study, the accuracy (ACC) and response time (RT) in the experimental trials of the Director and No-Director conditions were examined. In the Director condition, ACC scores indicated the degree to which the participant could take someone else’s visual perspective. RT scores revealed how quickly the participant correctly adopted the Director’s perspective and used the information to select the correct object. In the No-Director condition, ACC scores estimated the participant’s ability to follow the rule to ignore the items placed in the grey slots of the shelf. RT scores evaluated how quickly the participant performed the instruction. In both conditions, mean RT scores were computed from correct answers only.

The Youth Self-Report-YSR [49] was used to assess externalizing (including aggressive behaviors, attention problems, and delinquent behavior) and internalizing (including withdrawal, anxiety and depression, and somatic complains) problems in participants aged <18 years. For the participants aged ≥18 years, the Adult Self-Report-ASR [50] was used. Each of the 119 items in these instruments is evaluated on a 3-point scale, with 0 corresponding to “not true”, 1 to “sometimes true” and 2 to “very or often true”. The questionnaires were validated in a Francophone population (α>0.80).

#### Cognitive functioning

The French versions of the Vocabulary and the Block Design subtests of the Wechsler Intelligence Scale for Children–Fourth edition- WISC [51] or, for participants ≥18 years old, the Wechsler Adult Intelligence Scale–Third edition [52] was used to assess participants’ cognitive functioning. The Vocabulary subtest measures word knowledge, language development, and concept understanding. The Block Design subtest measures abstract visual information processing and visual problem-solving.

#### Psychopathic traits

The French version of the Youth Psychopathic Inventory- YPI [2] was used to assess psychopathic traits in the sample. The YPI evaluates three dimensions of psychopathy, each consisting of several subscales: an interpersonal dimension, assessing grandiose, manipulative behaviors, an affective dimension assessing callous-unemotional traits, and a dimension assessing impulsive, irresponsible behavior. The 50 items of the YPI are scored on a Likert scale from 1 “does not apply at all” to 4 “applies very well”.

Each participant was tested individually by a trained psychologist from our team. The IA were tested at the detention center’s facilities, in a private interview room, whereas the CA were tested our research unit. The protocol lasted for approximately one hour and a half. All the adolescents completed the full protocol except for two participants from the IA group, of whom one did not complete the Wechsler Intelligence Scale and the other did not complete the Youth Psychopathic Inventory.

### Data Analysis

Student’s *t*-tests were conducted to investigate differences in sample characteristics (age, externalizing and internalizing problems, psychopathic traits, and scores in the Vocabulary and Block Design subtests).

In order to investigate between-group differences in ER abilities, a multivariate analysis of variance (MANOVA) was conducted for the mean unbiased hit rate for the recognition of each emotion, with group (IA vs. CA) as between-subject variable. In order to investigate the results for the PT task, two-way mixed-model analysis of variance (ANOVA) was conducted for the ACC and RT scores with task condition (Director vs. No-Director) as a within-subject factor and group (IA vs. CA) as between-subject factor. Bivariate Spearman correlation analysis was used to investigate the relationship between performance in the GERT and the Director task and psychopathic traits in each group. Bivariate correlation analyses were also used to investigate the relationships between ER and ToM scores and the sample characteristics. Steiger’s Z (two-tailed) tests were conducted to test the differences in the correlation coefficients between the ER and the PT abilities between the two groups. A Bonferroni correction was applied for the number of correlations conducted, with significance accepted at *p* = 0.007. For all other statistical analyses, an alpha level of 0.050 was applied.

## Results and Discussion

### Sample Characteristics

Table 2 presents the results for the *t*-test analysis of the sample characteristics. In comparison with the CA group, the IA group had significantly lower scores for the Vocabulary (*t*(42) = 4.102, *p*<0.001, *d* = 1.26) and Block Design (*t*(42) = 3.748, *p* = 0.001, *d* = 1.15) subtests, significantly higher scores for the externalizing subscale in the YSR/ASR (*t*(43) = -5.026, *p*<0.000, *d* = -1.53), and significantly higher scores for the impulsive, irresponsible behavior subscale of the YPI (*t*(42) = -5.304, *p*<0.001, *d* = -1.63) and the YPI total score (*t*(42) = 2.670, *p*<0.001, *d* = 0.82). The groups did not differ for mean age (*t*(43) = -0.247, *p* = 0.806, *d* = -0.07) or for the internalizing subscale of the YSR/ASR (*t*(43) = -0.353, *p* = 0.726, *d* = -0.10).

**Table 2:** Sample characteristics results.

Variable	Community adolescents		Incarcerated adolescents	
	Mean	SD	Mean	SD
Age (years)	16.46	1.39	16.52	0.96
WISC/WAIS (Vocabulary)**	10.41	2.62	7.47	3.02
WISC/WAIS (Block design)**	10.95	2.21	7.42	4.2
YSR (externalizing)**	56.33	10.14	70.36	7.93
YSR (internalizing)	54.12	9.62	55.40	9.56
YPI (impulsive-irresponsible subscale)**	33.26	8.19	44.80	5.95
YPI (interpersonal problems subscale)	38.08	11.83	39.90	9.58
**YPI (callous-unemotional subscale)**	29.65	5.71	32.04	6.98

WISC, Wechsler Intelligence Scale for Children; WAIS, Wechsler Adult Intelligence Scale; YSR, Youth Self Report; YPI, Youth psychopathic Inventory.

** The mean difference between the two groups is significant at *p*<0.01.

### Emotion Recognition Task

**Fig 1:**
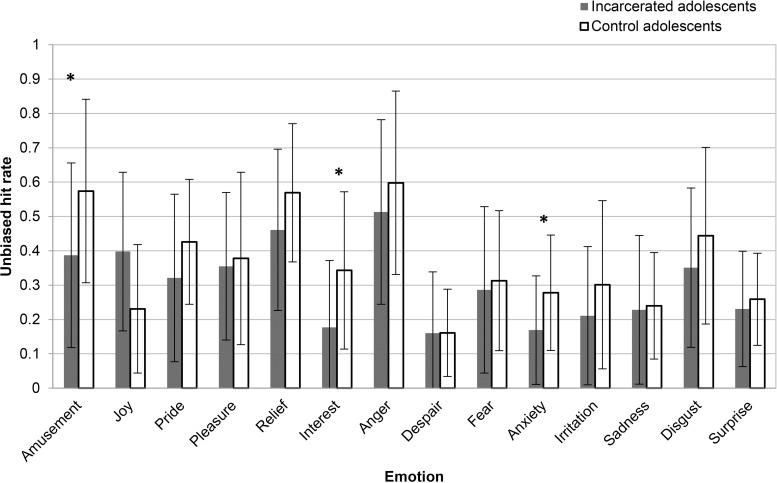
Means and standard deviations for the emotion recognition task for each group. * The mean difference between the two groups is significant at *p*<0.05.

The MANOVA revealed a significant effect of group on ER abilities (Pillais’ Trace *=* 0.507, *F*(14,30) = 2.205, *p* = 0.034, partial eta squared = 0.507, observed power = 0.878). Fig 1 presents the results from the ER task. Separate post hoc univariate ANOVAs on the recognition of each emotion indicated that the incarcerated participants scored significantly lower on recognition of anxiety (*F*(1, 43) = 6.00, *p* = 0.018, partial eta squared = 0.122, observed power = 0.668), amusement (*F*(1, 43) = 4.79, *p* = 0.034, partial eta squared = 0.100, observed power = 0.572), and interest (*F*(1, 43) = 7.59, *p* = 0.009, partial eta squared = 0.150, observed power = 0. 768) in comparison to the community adolescents. The differences between the two groups in the recognition of the other emotions did not reach the significance level (*p*>0.148).

### Perspective Taking Task

The results of the two-way mixed-model ANOVA conducted on the ACC scores in two Director task’s conditions, with group (IA vs. CA) as between factor, revealed a significant effect of group (*F*(1, 43) = 8.773, *p* = 0.005, partial eta squared = 0.169, observed power = 0.836), and a significant effect of condition (*F*(1, 43) = 84.656, *p*<0.001, partial eta squared = 0.663, observed power = 1), indicating that the IA group presented lower accuracy scores independently of the condition of the task. In addition, these results indicate that independently of the group, the ACC scores in the Director condition were lower than in the No-Director condition. The interaction effect between the group and the task’s conditions was not statistically significant (*F*(1, 43) = 0.210, *p* = 0.649, partial eta squared = 0.005).

The two-way mixed-model ANOVA conducted on the RT scores in the two conditions of the Director task, with group as between factor revealed no effect of group (*F*(1, 43) = 0.774 *p* = 0.385, partial eta squared = 0.020, observed power = 0.059), no effect of condition (*F*(1, 43) = 2.588 *p* = 0.116, partial eta squared = 0.064, observed power = 0.432), nor an interaction effect (*F*(1, 43) = 0.584 *p* = 0.450, partial eta squared = 0.015, observed power = 0.059).

### Relationship Between ER and PT Capacities and YPI Dimensions

In order to investigate whether the significant between-group differences in ER and PT abilities were driven by the group differences in WISC/WAIS subscale scores, we performed bivariate Pearson correlations between the scores for recognition of amusement, interest, and anxiety, the ACC scores in the Director and No-Director conditions, and the scores on the Vocabulary and Block Design subtests, for each group of adolescents. Bonferroni correction was applied for the number of correlations conducted, with significance being accepted at *p* = 0.007. The results revealed a negative correlation between the Block Design scores and the recognition of amusement in the CA group (*r* = -0.485, *p* = 0.019). However, this result did not reach the significance level after Bonferroni correction. No other significant correlation between these variables was revealed in either group.

To investigate the relationships between ER, PT, and the YPI dimensions, bivariate Spearman correlation analyses were conducted, in each group, between the scores in the recognition of amusement, anxiety, and interest, the ACC scores in the two conditions of the Director task, and the YPI scores in the impulsive-irresponsible subscale and in YPI total score. A Bonferroni correction was applied, with significance being accepted at *p* = 0.007. In the IA group, results indicated a significant positive correlation between the scores for recognition of interest and the ACC scores in the Director condition (*r* = 0.694, *p*<0.001), and a trend toward a significant correlation between the total YPI score and the scores for recognition of anxiety (*r* = 0.432, *p* = 0.051). No other significant correlations were found in either group.

Steiger’s Z test (two-tailed) examining the potential group difference in correlation coefficients between the recognition of interest and the ACC scores in the Director condition of the PT task revealed a trend-like significant difference (*z* = -1.94, *p* = 0.052).

## Discussion

The present study aimed to investigate the emotion recognition (ER) and visual perspective taking (PT) abilities in a group of incarcerated adolescents (IA) and a group of gender- and age-matched community adolescents (CA). We sought to overcome some of the methodological limitations of previous studies. More specifically, we used an ER task with high ecological validity, which presented an extended range of emotions portrayed by dynamic multimodal stimuli (video clips). In addition, to investigate PT, we employed a task that is sensitive to the development of PT abilities during adolescence and adulthood. We also aimed to explore how ER and PT capacities are related to each other, as well as to the different dimensions of psychopathy. The results indicate that, relative to the CA group, the IA group presented impairments in ER, specifically, the recognition of anxiety, amusement, and interest. In addition, the IA group showed lower accuracy (ACC) in both the Director and the No-Director conditions of the Director task. These results indicate that the apparent impairments in the recognition of interest in the IA group were positively correlated with the impairments in PT revealed by the Director task. Contrary to our expectations, no association was observed between the dimensions of psychopathy and the ER or the PT abilities.

Firstly, we will discuss the results of the emotion recognition task. In previous studies, antisocial behavior has been associated with impairments in the recognition of specific emotions, such as fear and sadness [1]. However, more recently, some authors have suggested that antisocial individuals present impairments in a wider range of emotions [7]. The results of the present study are in line with the theory of a general impairment in ER, rather than with the suggestion of impairments in recognition of specific emotions. Our results did not reveal any significant difference between the IA and CA groups in the recognition of basic emotions, consistent with previous studies that did not detect impairments in the recognition of these emotions in antisocial populations [53–55]. However, the IA group showed impaired recognition of anxiety, which is considered by some authors to be an expression of low-intensity fear [44]. This result is in line with previous studies reporting impairments in antisocial individuals in the recognition of emotions expressed at low intensities [56].

The IA group also presented lower recognition of amusement and interest than the CA group. These two emotions could be considered social emotions, as their recognition requires the inference of others’ mental states. Some authors have suggested that the development of the ability to recognize these emotions is sustained by the socialization process [56,57]. The apparent impairment in the recognition of amusement in the IA group result is in line with results of previous studies suggesting impairments in the recognition of happiness in antisocial populations [23,30,56,58]. Some authors have interpreted such results as indicating lack of exposure of antisocial individuals to the expression of happiness during their early social interactions, meaning that they lacked the opportunity to learn how to consistently identify this emotional expression during development [56].

With regard to the result for the recognition of interest, this emotion is considered to be associated with curiosity, exploration, and motivation to learn, and is implicated in establishing and maintaining interpersonal contact [59]. Some authors have suggested that the expression of interest plays a crucial role in the process of socialization [57,60]. By recognizing the other’s expression of interest, one identifies the important information in the environment and becomes more willing to assimilate it. Some authors postulate that adults use the expression of interest as an ostensive cue to indicate to a child with whom they are communicating that the knowledge contained within their communication is worth acquiring and retaining [60]. Other authors have suggested that interest might play an important role in the transmission of information even when it is not intentionally directed to the individual [57]. Observing the other’s interest in different aspects of the environment, one might identify the relevant elements for a group of people, and participate and share their experiences. Thus, recognizing others’ expressions of interest represents a crucial stage in the identification and acquisition of important information for a social group. Given that some authors suggest that socialization–the process of integrating socially relevant knowledge–restrains the manifestation of antisocial behavior [61], the impairments in the recognition of interest in the IA group in the present study might indicate challenges in socially adaptive behaviors and social practices. Further longitudinal studies are needed to explore the role of recognition of interest in the socialization process and in the emergence of antisocial behaviors.

With regard to PT abilities, results revealed that the IA group had lower ACC scores than the CA group in the Director condition and, contrary to our hypothesis, also in the No-Director condition. The results did not indicate any significant difference in RT between the groups, in the two conditions of the task. Lower ACC scores for the IA group in the PT condition are consistent with the results of previous studies that suggested impairments in the PT abilities in antisocial populations [62,63]. These results could be interpreted in the light of previous studies suggesting that the ability to infer intentions in others is correlated with social trust and serve to sustain positive social functioning [64]. Lower ACC scores for the IA group in the No-Director condition could be explained by impairments in executive function, given that, in this condition, the participants are required to follow a rule that they should ignore objects placed in some of the shelf slots. In line with the previous studies indicating a strong relationship between executive functions and PT, the impairments revealed by the Director condition of the PT task could be partially explained by the impairments in the executive functions, which might lead to the inability of the IA group to inhibit their own perspective, being more prone to egocentric biases. This hypothesis is in line with previous studies [65] indicating that the PT ability relies on executive functions when they are implicated in the self-other differentiation. The relationship between the executive functions and social cognition abilities, such as PT, is quite complex, and recent studies have provided preliminary evidence that the two processes are associated during the development [66]. However, a number of studies indicate that while these two processes are related, they are also independent: individuals presenting impairments in the social cognition can present intact executive functions [67], and individual showing impairment in executive functions can present high levels of social cognition [68–70]. In a related point, several studies suggest that executive functions significantly contribute to levels of intellectual quotient (IQ) [71]. Our results suggested that, compared to the CA, the IA group present lower scores in the subscales of WISC/WAIS. This result is in line with previous studies, which reported low IQ levels in antisocial individuals [72–74]. When we ran correlation analyses in order to test the relationship between the scores in WISC/WAIS subscales and the ACC scores in the Director and No Director conditions, we failed to observe significant results in either of the groups. While this issue requires further scrutiny in future studies, our results argue against the WISC/WAIS subscales scores playing a major role in the PT performances. This result is consistent with previous research suggesting that impairments in social-cognitive processes, such as PT, cannot be entirely explained by IQ levels [17,55,75,76]. The investigation of the relationships between the IQ, executive functions and PT in antisocial individuals is beyond the aims of the present study. However, future studies are needed to address this issue, using specific executive functions measures and a complete evaluation of intellectual abilities.

In our final analyses, we were interested in the relationship between PT and ER. Our results revealed a significant relationship between the recognition of interest and PT abilities in the IA group. No significant relationship was observed between ER and PT abilities in the CA group, or between the scores for ER and for the ACC scores in the No-Director condition in either of the groups. This result supports the hypothesis that the recognition of interest is closely related to the inference of others’ mental states in the IA group. Moreover, the association between the recognition of interest and the PT performances in the IA group could be interpreted in the light of the socialization theories discussed above. In the development of the processes of PT and the recognition of interest, the relationships between the child and the caregiver play an important role [60]. The impairments observed in the IA group in terms of PT abilities and recognition of interest might represent a consequence of early experience of an environment that was not rich in ostensive cues or did not consistently orient the individual to learn about mental states, leading to suboptimal social-cognitive functioning. Here, we may hypothesize that the inconsistencies in taking others’ perspective, combined with difficulties in attending to markers of interest in the behavior of others, may constitute social cognitive impairments that fail to moderate reactive violent reactions in antisocial, incarcerated youths. Additional longitudinal studies are needed to explore the relationship between the development of these two processes, as well as the role played by the relationship between the caregiver and child in this development.

Our results did not indicate a relationship between the ER or PT impairments and the dimensions of psychopathy, although in the IA group there was a trend toward a positive correlation between the total YPI score and the recognition of anxiety. These results are partially in line with previous studies that suggested that high psychopathy scores tended to be associated with better ER [24,53,77,78] and with no impairments in understanding the mental states in others [79]. The lack of relationship between ER or PT and the dimensions of psychopathy in the present study might be explained by the sample characteristics: in comparison with the CA group, the IA group presented higher scores for the impulsive-irresponsible dimension of psychopathy, but not for the interpersonal or unemotional dimensions.

Finally, the present study has several limitations that should be pointed out. First, our results require replication in a larger sample of IA. Second, the IA group had significantly higher scores than the CA group in the impulsive and irresponsible behavior subscale of the YPI, but not in the affective dimension of psychopathy. In future, it would be interesting to investigate how the different dimensions of psychopathy are related to ER and PT in antisocial adolescents. Another limitation that should be pointed out is the fact that not all the adolescents completed the semi-structured clinical interview; therefore we could not compare the two groups regarding their clinical characteristics. Finally, due to the small sample size and the limited available information about the crime history of the IA, we were unable to analyze the relationship between ER and ToM and different trajectories in antisocial behaviors. Given the fact that the onset of antisocial behavior in childhood is believed to be associated with a more negative outcome compared with onset in adolescence [3], it would be interesting to investigate whether these two types of trajectories are differently correlated to ER and PT abilities. Further longitudinal studies are needed to explore the relationships between these two processes and their role in the different trajectories of antisocial behavior.

## Ethical Approval

Written informed consent was obtained from all the participants and, for participants under 18 years old, also from their parents. The protocol was approved by the Institutional Review Board of the Department of Psychiatry of the University of Geneva Medical School.
